# Blood-Brain Barrier Disintegration in Growth-Restricted Fetuses with Brain Sparing Effect

**DOI:** 10.3390/ijms232012349

**Published:** 2022-10-15

**Authors:** Natalia Misan, Sławomir Michalak, Katarzyna Kapska, Krystyna Osztynowicz, Mariola Ropacka-Lesiak

**Affiliations:** 1 Department of Perinatology and Gynecology, Poznan University of Medical Sciences, 33 Polna Street, 60-535 Poznan, Poland; 2 Department of Neurochemistry and Neuropathology, Chair of Neurology, Poznan University of Medical Sciences, 49 Przybyszewskiego Street, 60-355 Poznan, Poland

**Keywords:** blood-brain barrier, brain injury, brain-sparing, fetal growth restriction, fetal hypoxia, intraventricular hemorrhage, tight junctions, tight junction proteins

## Abstract

The endothelial cells of the blood-brain barrier adhere closely, which is provided by tight junctions (TJs). The aim of the study was to assess the damage to the endothelial TJs in pregnancy, complicated by fetal growth restriction (FGR) and circulatory centralization (brain-sparing effect, BS). The serum concentrations of NR1 subunit of the N-methyl-D-aspartate receptor (NR1), nucleoside diphosphate kinase A (NME1), S100 calcium-binding protein B (S100B), occludin (OCLN), claudin-5 (CLN5), and zonula occludens protein – 1 (zo-1), and the placental expressions of OCLN, claudin-4 (CLN4), CLN5, and zo-1 were assessed with ELISA. The significantly higher serum NME1 concentrations and the serum CLN5/zo-1 index were observed in FGR pregnancy with BS, as compared to the FGR group without BS. The FGR newborns with BS were about 20 times more likely to develop an intraventricular hemorrhage (IVH) than the FGR infants without BS. The cerebroplacental ratio (CPR) allowed to predict the IVH in growth-restricted fetuses. The significantly lower placental CLN4 expression was observed in the FGR group with BS and who postnatally developed an IVH, as compared to the growth-restricted infants with BS without IVH signs. Pregnancy complicated by FGR and BS is associated with the destabilization of the fetal blood-brain barrier. The IVH in newborns is reflected in the inhibition of the placental CLN4 expression, which may be a useful marker in the prediction of an IVH among growth-restricted fetuses.

## 1. Introduction

FGR affects about 3 to 7% of pregnancies and is defined as a failure to achieve the genetically predicted growth [1,2]. FGR is related to an increased rate of stillbirth, neonatal mortality or morbidity [3,4], and the main factor contributing to these adverse outcomes, is a fetal brain injury. The neurological consequences include learning and attention difficulties, neurobehavioral impairments, cerebral palsy, epilepsy, and other cognitive disorders [5]. The mechanisms confused in FGR brain damage have been evaluated in preclinical studies concerned with excitotoxicity, oxidative stress, apoptotic degeneration, and neuroinflammation [6,7]. In hypoxic conditions, a cascade of cellular and biochemical events is activated, that lead to immediate or delayed cell death, with possible effects on immature neurons and neuroglia [7,8,9]. If the fetus is mature, its tolerance to chronic hypoxia is reduced, which results in circulatory centralization [1,10]. Initially, the adaptations to inadequate oxygen delivery cause an increase in cerebral, myocardial, and upper body blood flow, while perfusion of the kidneys, gastrointestinal tract, and lower extremities decreases. Circulatory centralization allows for the blood redistribution and enables the preferential delivery of nutrients and oxygen to the vital organs [11,12,13,14]. In the case of brain vasodilation, the cerebral perfusion is not homogeneous, and promotes particularly the basal ganglia. The blood supply of other brain regions, such as the frontal lobe, may therefore be modified, leading to an impaired neurodevelopment [15]. Eventually, as adaptive mechanisms are exhausted, the circulatory centralization does not provide sufficient protection against hypoxia for the developing fetal brain and may result in neurodevelopmental disorders [16,17]. 

The brain damage in FGR is a consequence of grey and white matter injury, as reported in clinical imaging studies [18,19]. Tolsa et al. found a reduced cortical grey matter volume of up to 28% in growth-restricted infants, in comparison to healthy term newborns [20]. Dubois et al. observed the delayed cortex development and gyrification in FGR infants after delivery [21]. These findings were confirmed up to one year-old children with neurological disabilities [19]. The post-mortem studies revealed a decreased neuronal cortex density [22]. The disturbed neuronal migration and proliferation, observed in the hippocampus and in the septo-hippocampal circuit in FGR, are supposed to be responsible for learning and memory dysfunction [9,23,24,25]. Moreover, the impaired neuronal connectivity and myelination were described in animal models of placental insufficiency [23,26], and in human prematurely born FGR infants [9,27,28]. Ramenghi et al. noticed a significant reduction in the myelination in neonates with FGR and BS during pregnancy, as compared to neonates with FGR and normal middle cerebral artery flows [29]. The magnetic resonance imaging revealed the delayed myelination and reduced posterior cerebral white matter volume, in the absence of demyelinating foci, in preterm infants with BS observed in pregnancy [6,29]. In addition, reduced volumes of the hippocampus, and cerebellum have been found, indicating these areas as particularly sensitive to hypoxia [6,30]. Moreover, the prematurely born FGR neonates had a lower global and a local neural connectivity, and a reduced cortico-basal ganglia association, mainly in the prefrontal cortex and limbic system, as compared to premature appropriate for gestational age neonates [6,31,32].

Infants with a prenatal diagnosis of late-onset FGR and circulatory centralization, born prematurely or at term, showed neurobehavioral impairments during the neonatal period, and at the age of two years. Similarly, neonates with an elevated umbilical artery pulsatility index during pregnancy, had a significantly poorer motor and cognitive development, when they were two years old, and at school age, as compared to infants born prematurely or at term without growth disorders [6,33,34]. Scherjon et al. reported no abnormal neurodevelopmental outcomes in three year old children with cardiac centralization, but at age of five years, the researchers observed a lower intelligence quotient, in comparison to the control group. In addition, the poorer outcomes in motor function, cognitive, and behavioral abilities have been observed in older children and adolescents, born prematurely with an umbilical artery reversed end diastolic flow during pregnancy [6,35,36]. However, cardiac centralization does not protect against neurodevelopmental disorders, which depend on the exposure to hypoxia, the severity of the FGR, and the pregnancy advancement at delivery [19,37]. Moreover, chronic hypoxia in prenatal life may lead to an increased blood-brain barrier permeability through the oxidative stress and inflammation, and induce a disruption of the integral proteins, resulting in this neurovascular unit breakdown [38].

The blood-brain barrier is formed by a monolayer of microvascular endothelial cells that line the cerebral capillaries, and contact with the vascular smooth muscle cells, pericytes, astrocytes, microglial cells, and neurons. These multicellular interactions, and the mutual transmission of the signals between them are defined as the neurovascular unit [39,40,41]. The brain endothelial cells adhere closely, which is provided by the TJs. The TJs are composed of transmembrane proteins (claudins, OCLN) and scaffolding proteins (zonula occludens proteins, zo) that anchor its brands to the actin cytoskeleton [42]. The blood-brain barrier disintegration is the main factor of cerebrovascular diseases and is characterized by the blood components infiltration, a changed transport, and a clearance of molecules into the central nervous system [43]. However, the interaction between the neurovascular unit cells is partially explored, and our knowledge of the fetal blood-brain barrier development remains incomplete. Moreover, there is no scientific data that report the effects of gestational hypoxia in growth-restricted fetuses, on vascular permeability, and the expression of the blood-brain barrier structural proteins. Therefore, the consequences of prenatal hypoxia on fetal growth and development can only be extrapolated in newborn studies, animal models, and adults with cerebral ischemia.

The aim of the study was to assess the damage to the endothelial TJs in a FGR pregnancy with BS. Moreover, the objective was to evaluate the blood-brain barrier permeability in growth-restricted fetuses with a hemodynamic redistribution, resulting in the appearance of neuronal injury markers in the maternal blood. Furthermore, the usefulness of the tight junction proteins (TJPs), and neuronal proteins in the prediction of neurological disorders in FGR with BS, was studied. The objectives were realized on the basis of serum neuronal protein measurements (NR1, NME1, S100B), serum TJPs concentrations (OCLN, CLN-5, zo-1, OCLN/zo-1 index, CLN-5/zo-1 index), and placental TJPs expressions (OCLN, CLN-4, CLN-5, zo-1).

## 2. Results

### 2.1. The Group Characteristics

The groups included adult women between 22 and 41 weeks of pregnancy. The studied groups were matched according to age, BMI, gravidity, and parity. The FGR with BS was diagnosed significantly earlier, as compared to the group without BS. The frequency of an early- and late-onset FGR diagnosis was comparable between the groups (Table 1). In the group with BS, there was a significant negative correlation between the serum NR1 levels and the gestational age at which the FGR was diagnosed (r = −0.35; *p* = 0.0250).

### 2.2. The Doppler Ultrasound Findings

The BS was diagnosed, on average, at 32 weeks’ gestation (Median: 32, Min: 24, Max: 39 weeks). The FGR stages were significantly more advanced in the group with BS. The growth-restricted fetuses with BS had a significantly lower EFW and EFW percentile, as compared to the group without a hemodynamic redistribution. The oligohydramnios was noticed statistically more often in the FGR pregnancies with the decreased CPR, as compared to the growth-restricted fetuses without BS. The Doppler blood flow velocimetry abnormalities, except for the DV PI and frequency of the UA REDF and the UV pulsations, were more severe in FGR with BS (Table 2).

### 2.3. The Serum Measurements and the Placental Expression

The significantly higher serum NME1 concentrations and the serum CLN5/zo-1 index were observed in FGR with BS, as compared to the FGR without BS. No differences in the serum concentrations of NR1, S100B, OCLN, CLN5, zo-1, or the serum OCLN/zo-1 ratio were found, between groups (Table 3). The placental expression of OCLN, CLN5, CLN4, and zo-1 was comparable between groups (Table 4).

### 2.4. The Neonatal Complications

The women diagnosed with FGR and BS delivered significantly earlier, as compared to the group without BS. In the FGR group with BS, the statistically higher incidence of fetal distress and cesarean section rate, was observed. A significantly lower birth weight was observed in the group of FGR neonates with BS, and the percentage of very low and extremely low birth weight was higher in this group in comparison to the FGR neonates without signs of BS. The newborns with BS were hospitalized significantly longer than the FGR infants without BS. As the circulatory centralization in FGR pregnancies was observed, the Apgar scores at the 1 and 5 min were significantly lower. The presence of an IVH was diagnosed statistically more often in the FGR pregnancies with BS. Among the fetuses with FGR and BS, 4.9% of intrauterine fetal deaths were observed. In the group with BS, adverse newborn outcomes were found significantly often, except for the rate of postpartum death (Table 5).

### 2.5. The Association between the Serum and Placental Measurements, and the Newborns’ Neurological Disorders

**Figure 1:**
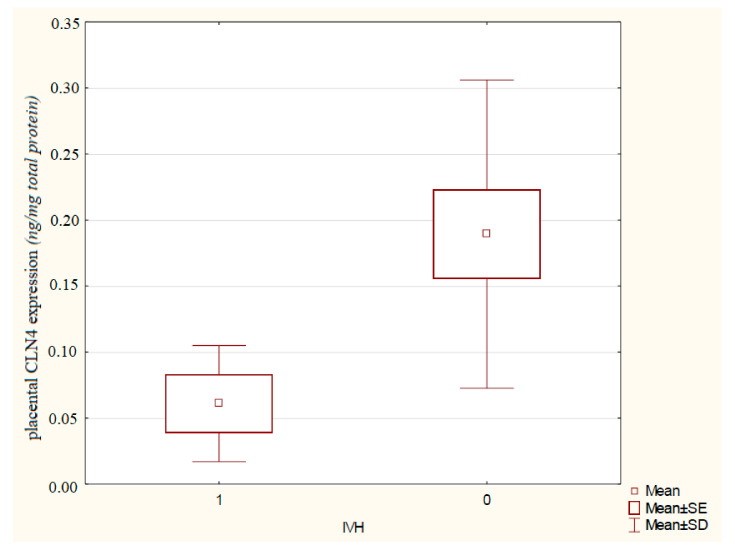
Comparison of the placental CLN4 expression in pregnancy complicated by fetal growth restriction with brain-sparing regarding the occurrence of an intraventricular hemorrhage in newborns.

The FGR newborns with BS were about 20 times more likely to develop an IVH than neonates with a normal CPR in pregnancy (OR = 21.52 [95% CI 1.18–389.39], *p* = 0.0377). The CPR allowed to predict the IVH in growth-restricted fetuses (cut-off: 0.76, sensitivity 100%, specificity 75%, PPV 0.28, NPV 1.00, AUC 0.923, *p* = 0.0002). In FGR pregnancies with BS and a postnatally developed IVH, the significantly lower placental CLN4 expression was observed (0.06 ± 0.04 vs. 0.19 ± 0.12 ng/mg total protein, *p* = 0.0391) as compared to the growth-restricted infants with BS without IVH signs (Figure 1). The placental CLN4 expression may be a useful marker in the prognosis of an IVH occurrence in growth-restricted newborns (cut-off: 20.82 ng/mg total protein, sensitivity 100%, specificity 83%, PPV 0.44, NPV 1.00, AUC 0.879, *p* = 0.0152).

## 3. Discussion

The brain injury in growth-restricted fetuses leads to short- and long-term consequences with limited possibilities for treatment. The brain endothelial function has an important role in many neurological diseases but there are no diagnostic tools to examine the fetal blood-brain barrier in clinical practice [44]. The TJPs that form the blood-brain barrier gave a promise as biomarkers in adults, and in animal models of brain injury [45,46], and may be tested as potential factors of fetal neuronal damage in prenatal life. Andersson et al. observed the time-dependent changes of the CLN5 and OCLN concentrations in the blood and cerebrospinal fluid after a hypoxic brain damage in neonatal rats. Moreover, the CLN5 levels in the cerebrospinal fluid were associated with the severity of the brain damage at 24 h after ischemia. Furthermore, the blood-brain barrier disintegration was detected at 6 and 24 h following the onset of hypoxic conditions [44]. Jiao et al. revealed the immunohistochemical time-dependent changes in the localization, and expression of CLN5, OCLN and zo-1, followed a middle cerebral artery occlusion, in rats. A significant decrease in the mean optical density of CLN5, OCLN and zo-1 was observed in the reperfusion groups, as compared to rats without a middle cerebral artery occlusion [47]. Su et al. observed that the treatment with a histone deacetylases inhibitor, in mice with an induced cerebral ischemia, increases the expression of zo-1, OCLN, and CLN5 in the brain endothelial cells, that improves the blood-brain barrier integrity [48]. Additionally, the hypoxic conditions and the glucose deprivation support the expression of the histone deacetylase 9, leading to a decrease of the zo-1, CLN5 and OCLN expressions [49]. Referring to the research from neurology, Kazmierski et al. found significantly higher levels of S100B, OCLN, and the CLN5/zo-1 ratio in patients with a clinical deterioration caused by a hemorrhagic transformation in ischemic stroke patients, as compared to those whose situation was not deteriorating. Moreover, the researchers noticed the associations between the circulating TJPs and the S100B concentration—a well-known marker of blood-brain barrier damage and concluded that the TJPs and the S100B protein may be useful in the screening of the hemorrhagic transformation in ischemic stroke patients. In this study increased CLN5/zo-1 ratio indicated clinical deterioration patients caused by hemorrhagic transformation of ischemic stroke. The increased CLDN5/ZO1 ratio was evaluated in the patients hospitalized, less than 6 h after the onset of a stroke, discriminated between the groups of ischemic stroke patients with and without a hemorrhagic transformation. As a predictor of a hemorrhagic transformation in ischemic stroke patients, the CLDN5/ZO1 ratio had the highest sensitivity and an even higher specificity than S100B [50]. Thus, the significance of the ratios between the more externally expressed CLN5 and internally expressed zo-1, was found clinically useful in ischemic strokes, as well as in our study. Moreover, the changes in such ratio indicate the rearrangement of the blood-brain barrier components exposed to noxious factors i.e., ischemia/hypoxia. So we concluded that the higher value of the CLN5/zo-1 ratio in FGR with BS, and the increased NME1 levels may further confirm its disorganization, both in the vascular and neuronal compartments. Pan et al. noticed that cerebral hypoxia induced an increase in the serum OCLN levels with a great increase at 4.5 h after the onset of ischemic conditions, which coexisted with the loss of OCLN from the hypoxia-affected cerebral micro vessels. Moreover, the serum OCLN concentrations stayed significantly higher than their output level within the first 24 h after the ischemic conditions [51]. Ma et al. reported the role of the epigenetic mechanisms mediated by microRNA, involved in the blood-brain barrier tightness [52]. Going further, Toyama et al. showed that miR-125-5p has a critical role in the brain endothelial integrity during inflammation. In this inflammatory response, there are involved, the specific mRNA targets of miR-125-5p, which downregulate CLN5 and CLN1, and cause the disruption of the adhesion molecules in the blood-brain barrier [53]. Moreover, Cai et al. revealed that the cerebral endothelial miR-144 downregulates CLN5, CLN12, OCLN, zo-1, zo-2, and zo-3 in a model of the blood-brain barrier permeability associated with brain tumors [54]. Similar to the cited studies, although the serum CLN5 concentrations did not differ in our study, the comparison was almost significant, which might suggest a tendency of this TJPs to decrease in FGR with BS. 

There are some data on the role of NME1 in the processes involved in neuronal development and brain damage, that come mainly from in vitro observations, and animal models [55,56]. From the point of our study, it is important that the cytosolic NME1 has been shown to interact with the components, and the regulators of the cytoskeleton, such as the actin-binding proteins, the intermediate filaments, and the enhancer structures (adherens junctions, desmosomes, focal adhesions) [57,58,59]. While most authors have focused on the role of the intracellular NME1, over time it has been observed in vitro that the extracellular protein can stimulate the growth of the dorsal root ganglion neurites. Wright et al. showed that the extracellular NME1 acts as a positive chemotactic signal that induces the neuronal growth bundles to move toward an environment with higher concentrations of NME1. A similar but more moderate effect was also observed in the absence of the nerve growth factor. In addition, greater axonal branching was found in the NME1-coated media, so it was hypothesized that the extracellular NME1 also plays a role in the central nervous system development [60,61]. Lööv et al. detected NME1 only in the culture of damaged neurons, suggesting a potential neuroprotective or regenerative function of this extracellular factor and a lack of its secretion under normal conditions [60,62]. Moreover, Lescuyer et al. observed an increase of NME1 in cerebrospinal fluid within 6 h of death, signaling that this protein may be a marker for neurodegenerative diseases [60,63]. In addition, Allard et al. identified NME1 as a sensitive and specific marker of strokes, and an increase of NME1 concentrations was observed within 3-6 h after the onset of symptoms [60,64]. To the best of our knowledge, the NME1 protein has not been studied in pregnancy complicated by FGR. The high value of the present study is to establish the role of NME1 as a biomarker in the prediction of the fetal circulatory centralization. The cut-off, with a sensitivity of 48% and a specificity of 92%, was set at 26.31 pg/mL. This concentration is lower than the threshold for the prediction of neurological complications in FGR neonates reported in our previous unpublished study. Considering the reports of the NME1 appearance (only in the culture of damaged neurons), and the fact that it was found in both groups, with and without blood flow centralization, it is reasonable to assume that BS does not protect against neuronal damage (which may begin much earlier). The NME1 concentration seems to be a prognostic factor to predict the optimal time of delivery in FGR pregnancies, to protect the fetus from an impaired neurodevelopment. Therefore, it is so important to find the markers of neuronal damage that will allow us to assess, as accurately as possible, the destabilization of the blood-brain barrier, to prevent neurodevelopmental disorders.

There are no data available on the role of NR1 in fetal brain damage, due to hypoxia in growth-restricted fetuses. At the mammalian central nervous system synapses, glutamate is the main neurotransmitter, which mediates in an excitatory transmission [65]. It has been shown that the glutamate excess can lead to various nervous system abnormalities. In traumatic brain injury or spinal cord damage, there is a sudden release of large amounts of this neurotransmitter from the neurons. The excitotoxicity in a traumatic brain injury has been confirmed in both animal models and human studies, and indicates the possible neuroprotective potential of the NMDA receptor antagonists in treatment [66,67,68]. Similar disturbances have been observed in ischemic stroke, during which the membrane protein pumps were deprived of energy, and the neurons lost the ability to maintain an ionic homeostasis. Their depolarization, lysis and/or self-destruction occur, similar to TBI, and results in an abnormal glutamate accumulation, and the prolonged activation of the synapses, mainly due to its impaired uptake by the astrocytes [69]. High glutamate levels are also important in the pathophysiology of many neurodegenerative disorders. A slow excitotoxicity has been identified as a pathogenetic factor in the progressive neuronal loss in Alzheimer’s disease, Huntington’s disease, Parkinson’s disease, multiple sclerosis, amyotrophic lateral sclerosis, and HIV-associated dementia. A prolonged exposure to moderate concentrations of glutamate, is suspected to cause the overactivation of the NMDA receptors, inducing neuronal apoptosis [65,67,70]. The immature brain is particularly susceptible to a hypoxia-induced excitotoxicity mediated by the NMDA receptors because of the high expression, and activity of the NMDA receptors at an early stage of development, the special role of the NMDA receptor-mediated neurotransmission system in the maturation and plasticity of the developing neurons, changes in the NMDA receptor configuration, and the neurotransmitter affinity after the exposure to noxious stimuli [71]. The animal models revealed that prenatal hypoxia caused a decrease in the NMDA receptors, and their glutamate- and glycine-dependent activation [72,73]. The lower brain white matter density, and the abnormal NMDA receptor subunit composition in the hippocampus of young rats with an induced FGR, due to a placental insufficiency was observed. Moreover, the reduction in the NR1 subunit levels, and the ratio of NR2A to NR2B were found [74]. Furthermore, Phillips et al. noticed a shortening of dendrites, and a decrease in the NR1 density in response to hypoxia [75]. According to the available literature, the NR1 concentrations in pregnancies complicated by FGR have not yet been studied in humans. In our study, there were no differences in the NR1 levels between the group with and without BS. In addition, there was no correlation between the NR1 levels, and perinatal complications. However, it was observed that the NR1 concentrations decreased significantly with the increasing gestational age, and the fetal maturity. This may confirm the previous reports of a high NMDA receptor expression, and activity during early fetal life [71]. Understanding the role of the NMDA receptor, and the balance between its various subunits, in a pregnancy complicated by FGR, requires further research. Perhaps one should pay attention to the role of its agonists, and antagonists. Because of the suspected protective potential of the NMDA antagonists, they could find use in cases of confirmed blood-brain barrier damage.

Gazzolo et al. published a series of papers, which pointed to the importance of the S100B protein in fetal neuronal damage. The authors analyzed FGR pregnancies, and divided the patients into three subgroups—(1) pregnancies without BS, and no abnormalities in a postpartum transtemporal ultrasound, (2) pregnancies with BS, and no abnormalities in a cranial sonography after delivery, (3) pregnancies with circulatory centralization, and abnormalities identified in a postpartum ultrasound neuroimaging. Similar to our unpublished data, Gazzolo et al. reported significantly higher S100B concentrations in the FGR pregnancies, as compared to the healthy pregnant women. However the observed S100B levels in both groups were much higher than noticed by us. Opposite to our findings, the S100B concentrations differed significantly between the FGR groups as follows: the 2nd group showed higher S100B levels than the 1st, and the 3rd group had higher S100B levels, as compared to the 1st, and to the 2nd. These results may indicate that the FGR is an independent factor related to neuronal damage, which intensifies with the occurrence of a hemodynamic redistribution. Even though we did not observe the significant differences in the S100B concentrations between FGR with and without signs of circulatory centralization. However, BS was prenatally observed in each neonate with a postnatally recognized IVH, as Gazzolo et al. previously reported. Moreover, the incidence of IVH was significantly higher in the FGR group with circulatory centralization, and an abnormal transcranial neuroimaging than in other FGR subgroups or in the control group [76]. Otherwise, our study revealed the frequency of an IVH in 17.1% of the FGR cases with BS, whereas it was not observed in patients without BS. Moreover, the newborns from the FGR group with BS were at greater risk of an IVH, more than 20 times higher, than the FGR neonates without BS. It points out that the blood flow centralization, considered as an adaptive mechanism that preserves the brain oxygen supply in hypoxic conditions, does not sufficiently protect against a neuronal injury. Moreover, the CPR predicted the IVH in FGR neonates with a sensitivity of 100%. Therefore, one may come to the conclusion that BS is related to the fetal blood-brain barrier breakdown, and thus the appearance of early neurological abnormalities available for physical examination, and neuroimaging.

Gazzolo et al. tried to determine, whether the S100B levels could be helpful in the detection of neurological abnormalities in FGR fetuses. Maternal S100B concentrations oscillated below the detection level for the method. The S100B concentrations in the umbilical artery were found to be significantly higher in pregnancies with FGR, compared to physiological gestation. In addition, the higher S100B protein levels in the umbilical artery were found in pregnancies with FGR and BS, as compared to the FGR group without signs of blood flow redistribution. Moreover, the S100B concentrations in the umbilical artery correlated negatively with the MCA PI index, and positively with the CPR [77], which may indicate that the Doppler blood flow abnormalities might be associated with the fetal blood-brain barrier injury. The study results provided evidence that the S100B protein level is elevated in the umbilical artery in growth-restricted fetuses, which correlates with cerebral hemodynamic, suggesting that it may be a biochemical marker of neuronal damage in the perinatal period. Maternal serum S100B levels, contrary to our results, were indeterminate in the FGR group, and in physiological pregnancy. It may be explained by the oscillation of the S100B concentrations below the sensitivity threshold detection, set as 200 pg/mL [77], which is about 10 times higher than the mean S100B levels in our study. 

There were no changes in the placental TJPs expression between the groups, which may indicate that the fetal blood-brain barrier breakdown may precede the placental insufficiency and the TJs disruption. This may also result from the predominance of first stage hemodynamic disturbances in our FGR study group, which is associated with a mild placental dysfunction [1]. Furthermore, it should be considered that the placental vessels show a positive immunostaining for zo-1 throughout gestation, whereas OCLN is detected mainly at term [78]. Because the rate of preterm births in our study exceeded 70% in the FGR group with BS, the OCLN concentrations may be not adequate. The placental CLN4 expression was significantly lower in the FGR group with an IVH, as compared to a FGR pregnancy without this complication. This might suggest that the disorganization of placental TJPs, contributing to the reduced endothelial cell tightness, may influence further blood flow deterioration leading to a placental insufficiency, and in turn, affect the fetal blood-brain barrier stability, and the observed changes in the CLN5/zo-1 ratio. Of note, no correlation was observed between the placental TJPs expression, and the neonatal complications, except for the neurological disorders. The placental expression of OCLN, CLN5, CLN4, and zo-1 also did not correlate with perinatal outcome. Thus, one can speculate that the changes in the placental CLN4 expression, and its association with an IVH in newborns, reflect the processes related to the fetal blood-brain barrier destabilization in FGR pregnancies. The analysis of the TJPs expression in FGR requires further studies with particular emphasis on the severity of the Doppler blood flow abnormalities. Because there are no scientific reports in the literature about the placental TJPs expression in FGR pregnancies, the results will be referred as preeclampsia, that has a similar pathogenesis, and is also related to a placental dysfunction. Contrary to our findings, Lievano et al. reported a decreased amount of CLN5 in preeclamptic placentae, but similar to us, the researchers observed no differences in the zo-1 immunostaining. That suggests leakier TJs in the endothelium of human placentae, possibly as a result of a decreased perfusion [79]. Itoh et al. detected the zo-1 protein in all human placental endothelial junctions, regardless of the pregnancy advancement, and the location in the vascular tree. The researchers suggested that zo-1, except for anchoring and signaling functions, may also mediate in the cell adhesion, acting as a link between the complex of cadherin/catenin, and the actin cytoskeleton [80]. Contradictory to our results, Pidoux et al. found a decrease in the zo-1 placental expression and concluded that it is related to the reduction of the human trophoblast cell-cell adhesion and differentiation [81].

However, the role of mechanisms involved in the integrity of the cerebrovascular network during gestational hypoxia remains widely unexplored. The blood-brain barrier permeability is a major factor, which determines the cause, progression, outcome, and therapeutic effectiveness of various neurological impairments in postnatal life. Therefore, the fetal programming of the blood-brain barrier permeability in FGR, and coexisting hypoxia pose a unique challenge to the research community in searching for the involved processes, the effective clinical treatment [38], and the biomarkers useful in the prediction of the neurological consequences, in order to prevent an impaired neurodevelopment. The blood-brain barrier tightness focuses mainly on TJs, however, the adherens junctions regulate cell-cell adhesion between the endothelial cells, contributing to the overall junction arrangement, and the blood-brain barrier integrity [82]. The knowledge about the processes involved in the fetal blood-brain barrier damage seems to be crucial for the management of FGR pregnancies. The placental hypoxia assessment, and its influence on the alterations in the endothelial adherens junctions, as well as the influence of oxidative stress, seem to be crucial. In addition, it should be clarified in detail, whether a disturbed maternal-placental exchange may lead to the breakdown of the fetal blood-brain barrier and result in the appearance of indicators of neuronal damage in the umbilical cord blood.

## 4. Methods and Materials

The study was carried out in the Department of Perinatology and Gynecology, Gynecological and Obstetric Hospital in cooperation with the Department of Neurology, Division of Neurochemistry and Neuropathology of the Medical University in Poznan. The project was positively approved by the Bioethics Committee (issue 667/15, 11 June 2015, annex issue 787/17, 22 June 2017). The research was performed in accordance with the Helsinki Declaration. The patients signed an informed consent for the serum and placental samples collection. 

### 4.1. The Studied Groups

The first group included 41 women with a prenatal diagnosis of FGR and BS. The second group was composed of 49 patients in a FGR pregnancy without BS. FGR was diagnosed, according to the Figueras and Gratacós criteria [1]. A detailed medical interview was taken, that was focused on the patient’s obstetric history, current pregnancy, chronic diseases, including any central nervous system pathology, or diseases that may affect the blood-brain barrier, and medications. Then, each woman underwent a gynecological examination, and a fetal ultrasound imaging with Doppler blood flow velocimetry (GE Voluson E10 BT18). The ultrasound measurements included the estimated fetal weight (EFW), the EFW percentile, the amniotic fluid index, the umbilical artery pulsatility index (UA PI), the presence of an umbilical artery absent end diastolic flow (UA AEDF) or an umbilical artery reversed end diastolic flow (UA REDF), the middle cerebral artery pulsatility index (MCA PI), the left uterine artery pulsatility index (LUtA PI), the right uterine artery pulsatility index (RUtA PI), the ductus venosus pulsatility index (DV PI), and umbilical venous (UV) pulsations. The cut-off for the diagnosis of early-and late-onset FGR was 32 weeks [83]. The EFW and EFW percentile were calculated according to the Hadlock formula, based on the head circumference (HC), the biparietal diameter (BPD), the abdominal circumference (AC), and the femur length (FL) [84,85]. The oligohydramnios and polyhydramnios were recognized according to the percentile charts of Magann et al. [86]. The CPR was calculated as the quotient of the MCA PI and UA PI, and the result was interpreted with reference to the Baschat and Gembruch percentile curves [87]. The uterine artery score (UAS) was assessed according to Sekizuka and Gudmundsson [88,89]. The FGR stage was determined, according to the Figueras and Gratacós criteria [90]. The birth weight was divided into three categories: small (1500–2500 g), very small (1000–1500 g), and extremely small (<1000 g) [91]. Metabolic acidosis was considered, according to the severity, into mild (pH 7.20–7.29), moderate (pH 7.10–7.19), severe (pH 7.00–7.09), and extremely severe (pH < 7.00) [92]. In case of the 1-min Apgar scores below 10, the 3-min assay was also given.

The exclusion criteria were as follows (1) maternal factors: undernutrition, nicotinism, alcohol consumption, drug abuse, treatment with warfarin, antiepileptic medications, antineoplastic drugs, folic acid antagonists, cyanotic heart defects, heart failure, severe asthma, chronic obstructive pulmonary disease, cystic fibrosis, pregestational and gestational diabetes mellitus, renal failure, nephrotic syndrome, kidney transplant status, hemodialysis therapy, systemic lupus erythematosus, antiphospholipid syndrome, Crohn’s disease, ulcerative colitis, severe anemia, inherited blood disorders, and uterine anomalies; (2) fetal causes: genetic diseases, congenital malformations, osteochondrodysplasias, congenital infections; (3) placental abnormalities: placenta previa, circumvallate placenta, placenta membranacea, placental infarction or villous thrombosis, hemangiomas, and other placental tumors. No clinical symptoms of primary or secondary central nervous system diseases or pathology were found in women included in the study.

### 4.2. Collection of Blood and Placental Samples 

The whole blood with a volume of 7.5 mL was withdrawn from the mother’s peripheral vein, and collected into three Monovette tubes without anticoagulant and then centrifuged for 10 min at 2700 × *g*. The serum was placed into Eppendorf probes and frozen at −80 °C. The external part of the placental plate, seized 25 cm^2^, was taken after delivery. Fresh tissue, without fixation, was frozen at −80 °C.

### 4.3. The Serum Measurements

The commercial ELISA kits were used to measure the serum concentration of NR1 (Human Glutamate [NMDA] receptor subunit zeta-1, GRIN1 ELISA Kit, MyBioSource, San Diego, CA, USA), NME1 (Human Nucleoside diphosphate kinase A, NME1 ELISA Kit, MyBioSource, San Diego, CA, USA) and S100B (S100B human ELISA Kit, DRG MedTek, Warsaw, Poland). Because there are no commercial tests to assess the serum levels of OCLN, zo-1, and CLN5 available, these TJPs were determined by an in-house ELISA method applied in the Department of Neurology, Division of Neurochemistry and Neuropathology, Medical University in Poznan. For the capture and detection of OCLN and zo-1, the rabbit anti-human (Occludin Polyclonal Antibody, Zymed, South San Francisco, CA, USA, AB_2533977, ZO-1 Polyclonal Antibody, Zymed, South San Francisco, CA, USA, AB_2533938) and mouse anti-human antibodies (Occludin Monoclonal Antibody (OC-3F10), Invitrogen, Waltham, MA, USA, AB_2533101, ZO-1 Monoclonal Antibody (ZO1-1A12), Invitrogen, Waltham, MA, USA, AB_2533147) were used. The CLN5 concentration was assessed by the use of mouse anti-human antibodies (Claudin 5 Monoclonal Antibody (4C3C2), Zymed, South San Francisco, CA, USA, AB_2533200) for the capture and rabbit anti-human antibodies (Claudin 5 Polyclonal Antibody, Abcam, Cambridge, UK, AB_2533157) in the detection. As secondary antibodies for OCLN and zo-1, goat anti-mouse IgG (Goat anti-Mouse IgG (H+L) Cross-Adsorbed Secondary Antibody, HRP, Invitrogen, Waltham, MA, USA, AB_2536527) was used. Likewise, the goat anti-rabbit IgG (H+L, HRP, Invitrogen, Waltham, MA, USA) were used in CLN5 serum determinations. The Substrate Reagent Pack (Substrate Reagent Pack, R&D Systems, Minneapolis, MN, USA) was applied in the analyzes. The recombinant proteins served as standards for OCLN (Recombinant Human Occludin GST (N-Term) Protein, Novus Biologicals, Littleton, CO, USA) and CLN5 (Recombinant Human Claudin-5 GST (N-Term) Protein, Novus Biologicals, Littleton, CO, USA). Because of the lack of the standard for zo-1, the relative units (RUs) were calculated on the basis of the measured optical density (OD) at 450 nm, as the quotient: OD for 10 samples/cut-off OD. The OD value was counted, based on the serum zo-1 concentrations of 48 control patients, and the 95th percentile was set as a threshold. The serum level of zo-1 was expressed as RU/mL, while the concentration of all other biochemical measurements was set in pg/mL. In the ELISA analyzes, the Nunc MaxiSorp^TM^ plates (ThermoFisher, Waltham, MA, USA), an automated RT-3100 microplate washer (Rayto Life and Analytical Sciences Co., Ltd., Shenzhen, China) and an ELx800 microplate reader (BioTek, Winooski, VT, USA) were used. The OCLN/zo-1 and CLN5/zo-1 ratios were calculated to evaluate the blood-brain barrier disintegration.

### 4.4. The Analyzis of Placental TJPs Expression

The placental expression of OCLN, zo-1, and CLN5 was measured by the immunoenzymatic ELISA method developed in the Department of Neurology, Division of Neurochemistry and Neuropathology, Medical University in Poznan. To determine the placental CLN4 expression, the commercial ELISA test was used (ELISA Kit for Claudin 4 (CLDN4), USCN Life Science, Wuhan, China). Initially, the placental tissues were homogenized in a buffer, consisting (in 1 L volume) of 150 millimoles (mM) NaCl (Sigma Aldrich, Saint Louis, MO, USA), 5 mM ethylenediaminetetraacetic acid—EDTA (Sigma Aldrich, Saint Louis, MO, USA), and a 50 mM Tris buffer solution (Sigma Aldrich, Saint Louis, MO, USA), to which a mixture of protease inhibitors (Protease Inhibitor Tablets For General Use, Sigma Aldrich, Saint Louis, MO, USA), and Triton X-100 (Sigma Aldrich, Saint Louis, MO, USA) was added. The solution’s final concentration was 1%. The mixed enzyme inhibitors included serine proteases inhibitors (fluorinated 4-(2-aminoethyl)benzenesulfonyl hydrochloride and aprotinin), aminopeptidases inhibitor (bestatin hydrochloride), trans-epoxysucinyl-L-leucylamido(4-guanidino)butane, inhibitor of metalloproteases (ethylenediaminetetraacetic acid), and an inhibitor of serine and cysteine proteases (leupeptin hemisulfate). Then the tissue homogenate was centrifuged for 15 min in Eppendorf tubes at 10,000 rpm, and the obtained filtrate was used for TJPs expression analyzes. All ELISA steps were carried out by the use of an automated microplate washer (RT-3100 Microplate Washer, Rayto Life and Analytical Sciences Co., Ltd., Shenzhen, China), and the results were recorded in an ELx800 microplate reader (Absorbance Microplate Reader, BioTek, Winooski, VT, USA). The TJPs expression was determined with the Lowry method [93] and given in ng/mg total protein. 

### 4.5. The Protocol of the Neurological Examination

All newborns were examined neurologically after delivery. The examination included the level of alertness, the cranial nerve function, the sensory and motor system control, and the presence of primitive reflexes. The infants born below 32 gestational weeks, underwent the first cranial sonography before they were three days old, and the second one between the 5th and 7th day of life. The IVH was diagnosed according to the Papile classification [94]. The subsequent imaging studies were dependent on the primary findings in the neuroimaging tests. An MRI was performed in cases of inconclusive or abnormal ultrasound scans. If the sonography imaging was normal, the MRI was carried out between 38 and 42 postconceptional weeks. The same protocol concerned newborns born between 32 and 35 weeks’ gestation. The infants, delivered after 35 weeks of pregnancy with an umbilical artery pH below 7.0 or an Apgar score less than or equal to 3 points, to whom therapeutic hypothermia was applied, underwent a transcranial Doppler ultrasound and an MRI between the 7th and 10th day of life. If the targeted temperature management was not obvious, the cranial sonography was performed up to the 3rd day of life, and then between the 5th and 7th days. If the newborns showed neurological abnormalities, the MRI was performed. The MRI scans allowed to confirm or exclude periventricular leukomalacia (PVL) [95,96]. 

### 4.6. Statistical Analysis

The data analysis was performed in Statistica StatSoft 13.1 (StatSoft, Kraków, Poland)** and MedCalc 20.113 (MedCalc Statistical Software Ltd., Katowice, Poland). The Kolmogorov–Smirnov, Lilliefors and Shapiro–Wilk tests were used to determine the normality of the distribution. If the assumptions were confirmed, the Student’s *t*-test was performed, otherwise the non-parametric Mann–Whitney U test was used. The Chi-squared and Fisher’s exact analyses were tested for data in a nominal scale. To measure the strength and direction of the association between the two ranked variables, the Spearman’s r correlation coefficient was assigned. The receiver operating characteristic (ROC) curves were calculated using the DeLong’s non-parametric method, and described as the cut-off value (cut-off), sensitivity, specificity, positive predictive value (PPV), negative predictive value (NPV), and the area under the curve (AUC). The threshold for the significance level was set at 0.05.

## 5. Conclusions

Pregnancy complicated by FGR with BS is associated with the destabilization of the fetal blood-brain barrier, as manifested by the significantly higher serum NME1 concentrations and the CLN5/zo-1 ratio. The fetal blood-brain barrier breakdown predates rather the placental insufficiency than it is a result of this, as reflected in the lack of differences in the placental expression of the tight junction proteins. In terms of neonatal complications after a FGR pregnancy with BS, the occurrence of an intraventricular hemorrhage was reflected in the changes of the analyzed biomarkers, namely in the inhibition of the placental CLN4 expression. The placental CLN4 expression may be a useful marker in the prediction of an IVH among growth-restricted fetuses. 

Further studies are needed to assess the stability of the blood-brain barrier in pregnancies complicated by FGR. The evaluation of the adherens junctions, and adhesion molecules could give us a more comprehensive understanding of the neurovascular unit integrity in growth-restricted fetuses. Moreover, the search for new markers of neuronal damage could allow us to better monitor FGR pregnancies, and predict impaired neurodevelopment, even before positive neuroimaging tests.

## Figures and Tables

**Table 1:** The demographic data of pregnant women with FGR according to the presence or absence of the brain sparing effect.

Characteristics	FGR Brain Sparing Effect (+) (*n* = 41)	FGR Brain Sparing Effect (−) (*n* = 49)	*p*
Age [years] (Mean ± SD)	29 ± 5	30 ± 5	0.5417
BMI at the first prenatal visit [kg/m^2^] (Median, Min-Max)	22.3 (16.2–42.0)	22.2 (15.2–38.1)	0.3837
The time of the FGR diagnosis			
(Median, Min-Max)	31 (22–39)	35 (27–40)	0.0006
early-onset (%)	34.2	51.0	0.1364
late-onset (%)	65.8	49.0	0.1364
Gravidity (Median, Min-Max)	1 (1–5)	1 (1–7)	0.3340
Parity (Median, Min-Max)	0 (0–3)	0 (0–4)	0.2661

**Table 2:** Ultrasound examination results in a FGR pregnancy in the presence or absence of the brain sparing effect.

Ultrasound Parameters	FGR Brain Sparing Effect (+) (*n* = 41)	FGR Brain Sparing Effect (−) (*n* = 49)	*p*
FGR staging			0.0009
I stage	68.3	96.0	
II stage	24.4	2.0	
III stage	4.9	0.0	
IV stage	2.4	2.0	
Estimated fetal weight [g] (Median, Min-Max)	1363 (439–2574)	2143 (671–2920)	0.0001
Percentile of estimated fetal weight (Median, Min-Max)	0.1 (0.1–9.0)	2.0 (0.1–9.0)	0.0001
Amniotic fluid index [cm]			
(Mean ± SD)	8.1 ± 3.8	9.3 ± 3.6	0.1293
oligohydramnion (%)	63.4	28.6	0.0009
polyhydramion (%)	2.4	0.0	0.4555
UA PI value			
(Median, Min-Max)	1.4 (0.7–3.0)	0.9 (0.6–1.4)	0.0001
abnormal UA PI (%)	78.1	10.2	<0.0001
UA AEDF (%)	24.4	2.0	0.0020
UA REDF (%)	4.9	0.0	0.2047
MCA PI value			
(Median, Min-Max)	1.1 (0.8–1.8)	1.6 (1.0–2.5)	0.0001
abnormal MCA PI (%)	73.2	18.4	<0.0001
CPR (Median, Min-Max)	0.9 (0.4–1.2)	1.7 (1.1–3.2)	0.0001
LUtA PI value			
(Median, Min-Max)	1.3 (0.5–3.2)	0.8 (0.5–3.0)	0.0017
abnormal LUtA PI (%)	58.5	18.4	<0.0001
RUtA PI value		0.8 (0.3–2.0)	0.0001
(Median, Min-Max)	1.3 (0.4–3.9)	12.2	<0.0001
abnormal RUtA PI (%)	58.5		
UAS score [points] (Median, Min-Max)	1 (0–4)	0 (0–3)	0.0001
DV PI value			
(Median, Min-Max)	0.5 (0.2–1.4)	0.6 (0.2–1.6)	0.7866
abnormal DV PI (%)	17.1	14.3	0.7163
UV pulsations (%)	2.4	0.0	0.4555

**Table 3:** Serum concentrations of the biochemical parameters in a FGR pregnancy in the presence or absence of the brain sparing effect.

Serum Measurements	FGR Brain Sparing Effect (+) (*n* = 41)	FGR Brain Sparing Effect (−) (*n* = 49)	*p*
NR1 [pg/mL] (Mean ± SD)	1121.79 ± 2263.20	1443.38 ± 3290.54	0.8291
NME1 [pg/mL] (Mean ± SD)	217.05 ± 951.86	23.04 ± 63.87	0.0261
S100B [pg/mL] (Mean ± SD)	29.07 ± 44.94	30.01 ± 37.10	0.6135
OCLN [pg/mL] (Mean ± SD)	18.68 ± 66.82	44.85 ± 145.66	0.8912
CLN5 [pg/mL] (Mean ± SD)	35.10 ± 101.37	107.63 ± 203.32	0.0529
zo-1 [RU/mL] (Mean ± SD)	1.95 ± 3.48	3.13 ± 5.32	0.9334
OCLN/zo-1 (Mean ± SD)	15.45 ± 68.28	6.98 ± 17.30	0.3979
CLN5/zo-1 (Mean ± SD)	190.51 ± 879.26	48.26 ± 112.54	0.0019

**Table 4:** Placental expression of the tight junction proteins in a FGR pregnancy in the presence or absence of the brain sparing effect.

Placental Expression	FGR Brain Sparing Effect (+) (*n* = 41)	FGR Brain Sparing Effect (−) (*n* = 49)	*p*
OCLN [ng/mg total protein] (Mean ± SD)	0.21 ± 0.16	0.15 ± 0.15	0.2095
CLN5 [ng/mg total protein] (Mean ± SD)	0.02 ± 0.03	0.01 ± 0.02	0.9712
CLN4 [ng/mg total protein] (Mean ± SD)	0.16 ± 0.11	0.16 ± 0.09	0.7916
zo-1 [RU/mg total protein] (Mean ± SD)	0.26 ± 0.16	0.23 ± 0.17	0.6419

**Table 5:** Perinatal outcomes of the growth-restricted fetuses with the presence or absence of the brain sparing effect.

Perinatal Outcomes	FGR Brain Sparing Effect (+) (*n* = 41)	FGR Brain Sparing Effect (−) (*n* = 49)	*p*
Gestational age at delivery [weeks] (Median, Min-Max)	34 (26–40)	39 (30–41)	<0.0001
Preterm birth (%)	73.2	16.3	<0.0001
Fetal distress (%)	65.9	34.7	0.0056
Mode of delivery (%)			
spontaneous	12.2	32.6	0.0396
cesarean section	87.8	58.7	0.0035
vacuum extraction	0.0	8.7	0.1188
forceps	0.0	0.0	-
Birth weight [g]			
(Median, Min-Max)	1560 (420–2740)	2540 (980–3080)	<0.0001
1500–2500 (%)	51.2	38.8	0.2890
1000–1500 (%)	14.6	2.0	0.0440
<1000 (%)	31.7	2.0	0.0002
Apgar score [points]			
(Median, Min-Max)			
1st min	9 (0–10)	10 (4–10)	0.0038
3rd min	8 (2–9)	8 (6–10)	0.3175
5th min	10 (4–10)	10 (7–10)	0.0010
pH			
(Median, Min-Max)			
venous	7.32 (7.19–7.40)	7.34 (7.01–7.46)	0.1661
arterial	7.28 (6.99–7.37)	7.27 (6.95–7.45)	0.7796
BE [mEq/L]			
(Median, Min-Max)			
Venous	−2.6 (−10.4–1.0)	−2.6 (−11.3–3.2)	0.8365
Arterial	−1.9 (−11.9–1.9)	−2.6 (−13.4–3.4)	0.1511
Metabolic acidosis (%)			
7.20–7.29	43.9	40.8	0.8319
7.10–7.19	9.8	14.3	0.7481
7.00–7.09	0.0	0.0	-
<7.0	2.4	2.0	1.0000
The hospitalization length [days] (Median, Min-Max)	20 (3–84)	5 (3–61)	<0.0001
Intraventricular hemorrhage (%)	17.0	0.0	0.0030
I grade	12.2	0.0	
II grade	2.4	0.0	
III grade	2.4	0.0	
IV grade	0.0	0.0	
Periventricular leucomalacia (%)	4.9	0.0	0.2047
Respiratory distress syndrome (%)	31.7	4.1	0.0005
I grade	17.1	2.0	0.0213
II grade	7.3	0.0	0.2427
III grade	4.9	2.0	0.5896
IV grade	2.4	0.0	0.4556
Respiratory failure (%)	34.2	8.2	0.0031
Bronchopulmonary dysplasia (%)	4.9	0.0	0.2047
Necrotizing enterocolitis (%)	4.9	0.0	0.2047
Intrauterine fetal death (%)	4.9	0.0	0.2094
Newborn death during hospitalization (%)	2.4	0.0	0.4555
Retinopathy (%)	9.8	0.0	0.0396

## Data Availability

The data presented in the study are available from the corresponding author upon reasonable request.

## Abbreviations

CLN4—claudin-4, CLN5—claudin-5, CLN5/zo-1 index—claudin-5/zonula occludens protein—1 index, FGR—fetal growth restriction, IVH—intraventricular hemorrhage, NME1—nucleoside diphosphate kinase A, NR1—NR1 subunit of the N-methyl-D-aspartate receptor, OCLN—occludin, OCLN/zo-1 index—occludin/zonula occludens protein—1 index, PVL—periventricular leucomalacia, S100B—S100 calcium-binding protein B, TJs—tight junctions, TJPs—tight junction proteins, zo-1—zonula occludens protein—1.

## References

[1] Figueras F., Gratacós E. (2014). Update on the diagnosis and classification of fetal growth restriction and proposal of a stage-based management protocol. Fetal Diagn. Ther..

[2] Sharma D., Shastri S., Sharma P. (2016). Intrauterine Growth Restriction: Antenatal and Postnatal Aspects. Clin. Med. Insights Pediatr..

[3] Jarvis S., Glinianaia S.V., Torrioli M.-G., Platt M.J., Miceli M., Jouk P.S., Johnson A., Hutton J., Hemming K., Hagberg G. (2003). Cerebral palsy and intrauterine growth in single births: European collaborative study. Lancet.

[4] Romo A., Carceller R., Tobajas J. (2009). Intrauterine growth retardation (IUGR): Epidemiology and etiology. Pediatr. Endocrinol. Rev..

[5] Wixey J.A., Chand K.K., Pham L., Colditz P.B., Bjorkman S.T. (2018). Therapeutic potential to reduce brain injury in growth restricted newborns. J. Physiol..

[6] Miller S.L., Huppi P.S., Mallard C. (2016). The consequences of fetal growth restriction on brain structure and neurodevelopmental outcome. J. Physiol..

[7] Rees S., Harding R., Walker D. (2011). The biological basis of injury and neuroprotection in the fetal and neonatal brain. Int. J. Dev. Neurosci..

[8] Poudel R., McMillen I.C., Dunn S.L., Zhang S., Morrison J.L. (2015). Impact of chronic hypoxemia on blood flow to the brain, heart, and adrenal gland in the late-gestation IUGR sheep fetus. Am. J. Physiol. Regul. Integr. Comp. Physiol..

[9] Colella M., Frérot A., Novais A.R.B., Baud O. (2018). Neonatal and Long-Term Consequences of Fetal Growth Restriction. Curr. Pediatr. Rev..

[10] Giussani D.A. (2016). The fetal brain sparing response to hypoxia: Physiological mechanisms. J. Physiol..

[11] Verburg B.O., Jaddoe V.W., Wladimiroff J.W., Hofman A., Witteman J.C., Steegers E.A. (2008). Fetal hemodynamic adaptive changes related to intrauterine growth: The Generation R Study. Circulation.

[12] Garcia-Canadilla P., Rudenick P.A., Crispi F., Cruz-Lemini M., Palau G., Camara O., Gratacos E., Bijnens B.H. (2014). A computational model of the fetal circulation to quantify blood redistribution in intrauterine growth restriction. PLoS Comput. Biol..

[13] MacDonald T.M., Hui L., Tong S. (2017). Reduced growth velocity across the third trimester is associated with placental insufficiency in fetuses born at a normal birthweight: A prospective cohort study. BMC Med..

[14] Cohen E., Wong F.Y., Horne R.S., Yiallourou S.R. (2016). Intrauterine growth restriction: Impact on cardiovascular development and function throughout infancy. Pediatr. Res..

[15] Hernandez-Andrade E., Figueroa-Diesel H., Jansson T., Rangel-Nava H., Gratacos E. (2008). Changes in regional fetal cerebral blood flow perfusion in relation to hemodynamic deterioration in severely growth-restricted fetuses. Ultrasound Obstet. Gynecol..

[16] Thompson L.P., Crimmins S., Telugu B.P., Turan S. (2015). Intrauterine hypoxia: Clinical consequences and therapeutic perspectives. Res. Rep. Neonatol..

[17] Malhotra A., Ditchfield M., Fahey M.C. (2017). Detection and assessment of brain injury in the growth-restricted fetus and neonate. Pediatr. Res..

[18] Esteban F.J., Padilla N., Sanz-Cortés M., de Miras J.R., Bargalló N., Villoslada P., Gratacós E. (2010). Fractal-dimension analysis detects cerebral changes in preterm infants with and without intrauterine growth restriction. Neuroimage.

[19] Padilla N., Falcón C., Sanz-Cortés M., Figueras F., Bargallo N., Crispi F., Eixarch E., Arranz A., Botet F., Gratacós E. (2011). Differential effects of intrauterine growth restriction on brain structure and development in preterm infants: A magnetic resonance imaging study. Brain Res..

[20] Tolsa C.B., Zimine S., Warfield S.K., Freschi M., Rossignol A.S., Lazeyras F., Hanquinet S., Pfizenmaier M., Hüppi P.S. (2004). Early alteration of structural and functional brain development in premature infants born with intrauterine growth restriction. Pediatr. Res..

[21] Dubois J., Benders M., Borradori-Tolsa C., Cachia A., Lazeyras F., Leuchter R.H.-V., Sizonenko S.V., Warfield S.K., Mangin J.F., Hüppi P.S. (2008). Primary cortical folding in the human newborn: An early marker of later functional development. Brain.

[22] Samuelsen G.B., Pakkenberg B., Bogdanović N., Gundersen H.J., Larsen J.F., Græm N., Laursen H. (2007). Severe cell reduction in the future brain cortex in human growth-restricted fetuses and infants. Am. J. Obstet. Gynecol..

[23] Khazardoost S., Ghotbizadeh F., Sahebdel B. (2019). Predictors of Cranial Ultrasound Abnormalities in Intrauterine Growth-Restricted Fetuses Born between 28 and 34 Weeks of Gestation: A Prospective Cohort Study. Fetal Diagn. Ther..

[24] Marsoosi V., Bahadori F., Esfahani F., Ghasemi-Rad M. (2012). The role of Doppler indices in predicting intra ventricular hemorrhage and perinatal mortality in fetal growth restriction. Med. Ultrason..

[25] Bernstein I.M., Horbar J.D., Badger G.J., Ohlsson A., Golan A. (2000). Morbidity and mortality among very-low-birth-weight neonates with intrauterine growth restriction. The Vermont Oxford Network. Am. J. Obstet. Gynecol..

[26] Gilbert W.M., Danielsen B. (2003). Pregnancy outcomes associated with intrauterine growth restriction. Am. J. Obstet. Gynecol..

[27] Guellec I., Marret S., Baud O. (2015). Intrauterine Growth Restriction, Head Size at Birth, and Outcome in Very Preterm Infants. J. Pediatr..

[28] Padilla N., Perapoch J., Carrascosa A., Acosta-Rojas R., Botet F., Gratacós E. (2010). Twelve-month neurodevelopmental outcome in preterm infants with and without intrauterine growth restriction. Acta Paediatr..

[29] Ramenghi L.A., Martinelli A., de Carli A. (2011). Cerebral maturation in IUGR and appropriate for gestational age preterm babies. Reprod. Sci..

[30] Lodygensky G.A., Seghier M.L., Warfield S.K. (2008). Intrauterine growth restriction affects the preterm infant’s hippocampus. Pediatr. Res..

[31] Batalle D., Eixarch E., Figueras F. (2012). Altered small-world topology of structural brain networks in infants with intrauterine growth restriction and its association with later neurodevelopmental outcome. Neuroimage.

[32] Fischi-Gómez E., Vasung L., Meskaldji D.E. (2015). Structural Brain Connectivity in School-Age Preterm Infants Provides Evidence for Impaired Networks Relevant for Higher Order Cognitive Skills and Social Cognition. Cereb. Cortex.

[33] Morsing E., Asard M., Ley D., Stjernqvist K., Marsál K. (2011). Cognitive function after intrauterine growth restriction and very preterm birth. Pediatrics.

[34] Vossbeck S., de Camargo O.K., Grab D., Bode H., Pohlandt F. (2001). Neonatal and neurodevelopmental outcome in infants born before 30 weeks of gestation with absent or reversed end-diastolic flow velocities in the umbilical artery. Eur. J. Pediatr..

[35] Scherjon S., Briët J., Oosting H., Kok J. (2000). The discrepancy between maturation of visual-evoked potentials and cognitive outcome at five years in very preterm infants with and without hemodynamic signs of fetal brain-sparing. Pediatrics.

[36] Scherjon S.A., Oosting H., Smolders-DeHaas H., Zondervan H.A., Kok J.H. (1998). Neurodevelopmental outcome at three years of age after fetal ‘brain-sparing’. Early Hum. Dev..

[37] Baschat A.A. (2014). Neurodevelopment after fetal growth restriction. Fetal Diagn. Ther..

[38] Herrera E.A., González-Candia A. (2021). Gestational Hypoxia and Blood-Brain Barrier Permeability: Early Origins of Cerebrovascular Dysfunction Induced by Epigenetic Mechanisms. Front. Physiol..

[39] Abbott N.J., Rönnbäck L., Hansson E. (2006). Astrocyte-endothelial interactions at the blood-brain barrier. Nat. Rev. Neurosci..

[40] Chow B.W., Gu C. (2015). The molecular constituents of the blood-brain barrier. Trends Neurosci..

[41] Banks W.A. (2016). From blood-brain barrier to blood-brain interface: New opportunities for CNS drug delivery. Nat. Rev. Drug Discov..

[42] Gonzalez-Candia A., Rogers N.K., Castillo R.L., Heinbockel T., Zhou Y. (2020). Blood-Brain Barrier Dysfunction in the Detrimental Brain Function. Connectivity and Functional Specialization in the Brain.

[43] Zhao Z., Nelson A.R., Betsholtz C., Zlokovic B.V. (2015). Establishment and Dysfunction of the Blood-Brain Barrier. Cell.

[44] Andersson E.A., Mallard C., Ek C.J. (2021). Circulating tight-junction proteins are potential biomarkers for blood-brain barrier function in a model of neonatal hypoxic/ischemic brain injury. Fluids Barriers CNS.

[45] Robinson B.D., Tharakan B., Lomas A., Wiggins-Dohlvik K., Alluri H., Shaji C.A., Jupiter D., Isbell C.L. (2020). Exploring blood-brain barrier hyperpermeability and potential biomarkers in traumatic brain injury. Proc. Bayl. Univ. Med. Cent..

[46] Shan R., Szmydynger-Chodobska J., Warren O.U., Mohammad F., Zink B.J., Chodobski A. (2016). A New Panel of Blood Biomarkers for the Diagnosis of Mild Traumatic Brain Injury/Concussion in Adults. J. Neurotrauma.

[47] Jiao X., He P., Li Y., Fan Z., Si M., Xie Q., Chang X., Huang D. (2015). The role of circulating tight junction proteins in evaluating blood brain barrier disruption following intracranial hemorrhage. Dis. Markers.

[48] Su L., Liang D., Kuang S.Y., Dong Q., Han X., Wang Z. (2020). Neuroprotective mechanism of TMP269, a selective class IIA histone deacetylase inhibitor, after cerebral ischemia/reperfusion injury. Neural Regen. Res..

[49] Shi W., Wei X., Wang Z., Han H., Fu Y., Liu J., Zhang Y., Guo J., Dong C., Zhou D. (2016). DAC9 exacerbates endothelial injury in cerebral ischaemia/reperfusion injury. J. Cell Mol. Med..

[50] Kazmierski R., Michalak S., Wencel-Warot A., Nowinski W.L. (2012). Serum tight-junction proteins predict hemorrhagic transformation in ischemic stroke patients. Neurology.

[51] Pan R., Yu K., Weatherwax T., Zheng H., Liu W., Liu K.J. (2017). Blood Occludin Level as a Potential Biomarker for Early Blood Brain Barrier Damage Following Ischemic Stroke. Sci. Rep..

[52] Ma F., Zhang X., Yin K.J. (2020). MicroRNAs in central nervous system diseases: A prospective role in regulating blood-brain barrier integrity. Exp. Neurol..

[53] Toyama K., Spin J.M., Tsao P.S. (2017). Role of microRNAs on Blood Brain Barrier Dysfunction in Vascular Cognitive Impairment. Curr. Drug Deliv..

[54] Cai W., Zhang K., Li P., Zhu L., Xu J., Yang B., Hu X., Lu Z., Chen J. (2017). Dysfunction of the neurovascular unit in ischemic stroke and neurodegenerative diseases: An aging effect. Ageing Res. Rev..

[55] Anantha J., Goulding S.R., Wyatt S.L., Concannon R.M., Collins L.M., Sullivan A.M., O’Keeffe G.W. (2020). STRAP and NME1 Mediate the Neurite Growth-Promoting Effects of the Neurotrophic Factor GDF5. iScience.

[56] Anantha J., Goulding S.R., Tuboly E., O’Mahony A.G., Moloney G.M., Lomansey G., McCarthy C.M., Collins L.M., Sullivan A.M., O’Keeffe G.W. (2022). NME1 Protects Against Neurotoxin-, α-Synuclein- and LRRK2-Induced Neurite Degeneration in Cell Models of Parkinson’s Disease. Mol. Neurobiol..

[57] Lombardi D., Sacchi A., D’Agostino G., Tibursi G. (1995). The association of the Nm23-M1 protein and beta-tubulin correlates with cell differentiation. Exp. Cell Res..

[58] Otero A.S. (2000). NM23/nucleoside diphosphate kinase and signal transduction. J. Bioenerg. Biomembr..

[59] Roymans D., Willems R., Vissenberg K. (2000). Nucleoside diphosphate kinase beta (Nm23-R1/NDPKbeta) is associated with intermediate filaments and becomes upregulated upon cAMP-induced differentiation of rat C6 glioma. Exp. Cell Res..

[60] Romani P., Ignesti M., Gargiulo G., Hsu T., Cavaliere V. (2018). Extracellular NME proteins: A player or a bystander?. Lab. Investig..

[61] Wright K.T., Seabright R., Logan A. (2010). Extracellular Nm23H1 stimulates neurite outgrowth from dorsal root ganglia neurons in vitro independently of nerve growth factor supplementation or its nucleoside diphosphate kinase activity. Biochem. Biophys. Res. Commun..

[62] Lööv C., Shevchenko G., Geeyarpuram Nadadhur A. (2013). Identification of injury specific proteins in a cell culture model of traumatic brain injury. PLoS ONE.

[63] Lescuyer P., Allard L., Zimmermann-Ivol C.G. (2004). Identification of post-mortem cerebrospinal fluid proteins as potential biomarkers of ischemia and neurodegeneration. Proteomics.

[64] Allard L., Burkhard P.R., Lescuyer P. (2005). PARK7 and nucleoside diphosphate kinase A as plasma markers for the early diagnosis of stroke. Clin. Chem..

[65] Dobrek L., Thor P. (2011). Glutamate NMDA receptors in pathophysiology and pharmacotherapy of selected nervous system diseases. Postepy Hig. Med. Dosw..

[66] Kemp J.A., McKernan R.M. (2002). NMDA receptor pathways as drug targets. Nat. Neurosci..

[67] Muir K.W. (2006). Glutamate-based therapeutic approaches: Clinical trials with NMDA antagonists. Curr. Opin. Pharmacol..

[68] Shohami E., Biegon A. (2014). Novel approach to the role of NMDA receptors in traumatic brain injury. CNS Neurol. Disord. Drug Targets.

[69] Hoyte L., Barber P.A., Buchan A.M., Hill M.D. (2004). The rise and fall of NMDA antagonists for ischemic stroke. Curr. Mol. Med..

[70] Lipton S.A. (2007). Pathologically-activated therapeutics for neuroprotection: Mechanism of NMDA receptor block by memantine and S-nitrosylation. Curr. Drug Targets.

[71] Waters K.A., Machaalani R. (2004). NMDA receptors in the developing brain and effects of noxious insults. Neurosignals.

[72] Tingley W.G., Ehlers M.D., Kameyama K. (1997). Characterization of protein kinase A and protein kinase C phosphorylation of the N-methyl-D-aspartate receptor NR1 subunit using phosphorylation site-specific antibodies. J. Biol. Chem..

[73] Mishra O.P., Delivoria-Papadopoulos M. (1992). Modification of modulatory sites of NMDA receptor in the fetal guinea pig brain during development. Neurochem. Res..

[74] Schober M.E., McKnight R.A., Yu X., Callaway C.W., Ke X., Lane R.H. (2009). Intrauterine growth restriction due to uteroplacental insufficiency decreased white matter and altered NMDAR subunit composition in juvenile rat hippocampi. Am. J. Physiol. Regul. Integr. Comp. Physiol..

[75] Phillips T.J., Scott H., Menassa D.A. (2017). Treating the placenta to prevent adverse effects of gestational hypoxia on fetal brain development. Sci. Rep..

[76] Gazzolo D., Marinoni E., Di Iorio R. (2006). High maternal blood S100B concentrations in pregnancies complicated by intrauterine growth restriction and intraventricular hemorrhage. Clin. Chem..

[77] Gazzolo D., Marinoni E., di Iorio R., Lituania M., Bruschettini P.L., Michetti F. (2002). Circulating S100beta protein is increased in intrauterine growth-retarded fetuses. Pediatr. Res..

[78] Marzioni D., Banita M., Felici A., Paradinas F.J., Newlands E., de Nictolis M., Mühlhauser J., Castellucci M. (2001). Expression of ZO-1 and occludin in normal human placenta and in hydatidiform moles. Mol. Hum. Reprod..

[79] Liévano S., Alarcón L., Chávez-Munguía B., González-Mariscal L. (2006). Endothelia of term human placentae display diminished expression of tight junction proteins during preeclampsia. Cell Tissue Res..

[80] Itoh M., Nagafuchi A., Moroi S., Tsukita S. (1997). Involvement of ZO-1 in cadherin-based cell adhesion through its direct binding to alpha catenin and actin filaments. J. Cell Biol..

[81] Pidoux G., Gerbaud P., Gnidehou S., Grynberg M., Geneau G., Guibourdenche J., Carette D., Cronier L., Evain-Brion D., Malassiné A. (2010). ZO-1 is involved in trophoblastic cell differentiation in human placenta. Am. J. Physiol. Cell Physiol..

[82] Li W., Chen Z., Chin I., Chen Z., Dai H. (2018). The Role of VE-cadherin in Blood-brain Barrier Integrity under Central Nervous System Pathological Conditions. Curr. Neuropharmacol..

[83] Gordijn S.J., Beune I.M., Thilaganathan B., Papageorghiou A., Baschat A.A., Baker P.N., Silver R.M., Wynia K., Ganzevoort W. (2016). Consensus definition of fetal growth restriction: A Delphi procedure. Ultrasound Obstet. Gynecol..

[84] Hadlock F.P., Harrist R.B., Martinez-Poyer J. (1991). In utero analysis of fetal growth: A sonographic weight standard. Radiology.

[85] Hadlock F.P., Deter R.L., Harrist R.B., Park S.K. (1984). Estimating fetal age: Computer-assisted analysis of multiple fetal growth parameters. Radiology.

[86] Magann E.F., Sanderson M., Martin J.N., Chauhan S. (2000). The amniotic fluid index, single deepest poecket and two diameter poeket in normal pregnancy. Am. J. Obstet. Gynecol..

[87] Baschat A.A., Gembruch U. (2003). The cerebroplacental Doppler ratio revisited. Ultrasound Obstet. Gynecol..

[88] Sekizuka N., Hasegawa I., Takakuwa K., Tanaka K. (1997). Scoring of uterine artery flow velocity waveform in the assessment of fetal growth restriction and/or pregnancy induced hypertension. J. Matern. Fetal Investig..

[89] Gudmundsson S., Korszun P., Olofsson P., Dubiel M. (2003). New score indicating placental vascular resistance. Acta Obstet. Gynecol. Scand..

[90] Figueras F., Gratacos E. (2014). Stage-based approach to the management of fetal growth restriction. Prenat. Diagn..

[91] Szczapa J., Wojsyk-Banaszak I., Dobrzańska A., Ryżko J. (2014). Wcześniactwo. Pediatria. Podręcznik do Lekarskiego Egzaminu Końcowego i Państwowego Egzaminu Specjalizacyjnego.

[92] Korones S.B. (1981). High Risk Newborn Infants. The Basis for Intensive Nursing Care.

[93] Lowry O.H., Rosebrough N.J., Farr A.L., Randall R.J. (1951). Protein measurement with the Folin phenol reagent. J. Biol. Chem..

[94] Papile L.A., Burstein J., Burstein R., Koffler H. (1978). Incidence and evolution of subependymal and intraventricular hemorrhage: A study of infants with birth weights less than 1500 gm. J. Pediatr..

[95] Baker L.L., Stevenson D.K., Enzmann D.R. (1988). End-stage periventricular leukomalacia: MR evaluation. Radiology.

[96] Barkovich A.J., Truwit C.L. (1990). Brain damage from perinatal asphyxia: Correlation of MR findings with gestational age. AJNR Am. J. Neuroradiol..

